# Associations of risk factors and the number of risk factors with the classification, GMFCS level and comorbidities with cerebral palsy: a retrospective study

**DOI:** 10.1186/s12887-024-05156-5

**Published:** 2024-12-19

**Authors:** Chao Gong, Pei Zeng, Beibei Lian, Jiawei Li, Jiahao Liu, Yuanyuan Liu, liya Fang, Huiling Tian, Luchuan Wang, Zhimei Jiang, Jin Guo, Shaobo Zhou

**Affiliations:** 1 College of Rehabilitation Medicine, Jiamusi University, Jiamusi, Heilongjiang China; 2 Linyi Maternal and Child Health Hospital, Linyi, Shandong China; 3 Jiamusi University Affiliated No. 3 Hospital, Jiamusi, Heilongjiang China; 4 Jiamusi University Affiliated No. 1 Hospital, Jiamusi, Heilongjiang China; 5 Faculty of Engineering and Science, University of Greenwich, Medway Campus Central Avenue, ChathamMaritime Kent, ME4 4TB England

**Keywords:** Cerebral palsy, Classification, GMFCS, Intellectual disability, Epilepsy, Hearing impairment, Retrospective study, Northeast China

## Abstract

**Objective:**

The objective was to explore the characteristics of risk factors in children with cerebral palsy (CP), focusing on the effects of single risk factors and the number of risk factors on the classification, GMFCS level, and comorbidities of children with CP.

**Methods:**

The medical records of children with CP hospitalized from 2015 to 2023 were reviewed. The effects of nine risk factors, such as hyperbilirubinemia, asphyxia, and HIE, on the classification, GMFCS level and comorbidities of children with CP were studied.

**Results:**

In Part 1, among the 536 children with CP, 476 (88.8%) had obvious risk factors. Preterm birth and/or low birth weight were the most common risk factor (243 cases (45.3%)). CP combined with two risk factors was the most common, with 147 cases (27.4%). In Part 2, neonatal seizures were associated with epilepsy, and HIE and hyperbilirubinemia were associated with intellectual disability. Asphyxia was associated with high GMFCS levels and mixed CP. Preterm birth and/or low birth weight was associated with spastic diplegia, and hyperbilirubinemia was associated with involuntary movement. In Part 3, the number of risk factors in children with CP with epilepsy and/or hearing impairment seemed to be lower, but those with spastic quadriplegia were more likely to have more risk factors (≥ 4). In the six groups with 1–6 risk factors, intellectual disability and a GMFCS level ≥ level IV were more common in the various risk factor groups, but spastic hemiplegia and ataxia were less common.

**Conclusion:**

Most children with CP have apparent risk factors, and the combination of two risk factors is relatively common. Preterm birth/low birth weight is the most common risk factor. The analysis of single risk factors revealed that the risk factors were related to the classification, GMFCS level and comorbidities. This correlation is consistent with the current research. Risk factors were more common in children with severe CP, high GMFCS levels, spastic quadriplegia, and intellectual disability.

## Introduction

Cerebral palsy (CP) is a group of persistent motor and postural developmental disorders and limited activity syndromes caused by nonprogressive brain damage in developing foetuses or infants [1, 2]. In children with CP, motor disorders are often linked with sensory, perceptual, cognitive, communication, and behavioural disorders, as well as epilepsy and secondary musculoskeletal problems [1, 2].

On the basis of population-based studies conducted globally, CP is estimated to have a prevalence of 0.1% to 0.4% [3]. In 12 provinces and cities in China, the prevalence of CP among children aged 1–6 years is reported to be 2.46 cases per 1000 children, leading to significant familial and social burdens, as well as increased utilization of medical resources [4]. Currently, the complex causes and development of CP remain unclear. The high-risk factors for CP are diverse and multifaceted [5]. These risk factors can occur before, during, or after birth over an extended period. Additionally, these risk factors interact with each other, forming a complex network that contributes to the development of the condition [6]. Regrettably, previous research on the risk factors for CP in children has been somewhat limited in scope, often focusing solely on birth and foetal factors [7]. For example, one study highlighted that preterm birth, asphyxia, and hyperbilirubinemia significantly increase the risk of CP, with perinatal and maternal factors frequently being overlooked [8]. Additionally, the Gross Motor Function Classification System (GMFCS) level in children with CP directly impacts their quality of life and daily functioning [2].

Before conducting this study, we gathered cross-sectional data on comorbidities in CP patients in China and performed a meta-analysis. The analysis revealed that 79.7% of CP patients had one or more comorbidities. Specifically, 17.9% of the CP patients had epilepsy, 58.0% had an intellectual disability, 48.0% had a language disability, 17.2% had a hearing disability, and 23.1% had a visual disability [9]. The relationships between comorbidities and the characteristics, severity, and risk factors for brain injury are important in the medical field. Identifying or updating the risk factors for paediatric CP can help in the development of preventive interventions. There are few studies on the risk factors for CP in children in China, especially in Northeast China, which is relatively cold and economically underdeveloped. Additionally, there is a lack of research on how the number of risk factors affects CP classification, the GMFCS level, and comorbidities. To address this gap, we conducted a retrospective analysis to examine the influence of individual risk factors and the number of risk factors on the classification, GMFCS level, and comorbidities of children with CP in a special children's hospital in Heilongjiang Province. We calculated the odds ratio (OR) and 95% confidence interval (CI) to provide a scientific basis for preventing and managing infantile CP.

## Methods

### Participants

The Jiamusi University Affiliated No. 3 Hospital, located in Jiamusi (47°N, 130°E), Heilongjiang Province, is one of the largest hospitals specializing in children's rehabilitation in Northeast China. Most children with CP in Heilongjiang Province receive rehabilitation treatment at our hospital, which is representative of the prevalence of CP in children in this area. The sample collection period was from January 2015 to June 2023. We retrospectively collected data on the risk factors recorded before and during the children's hospitalizations. These risk factors were recorded in medical records on the basis of patient/family self-reports or previous medical records. This study was approved by the Ethics Committee of Jiamusi University (No. jmsukf-2023012). Owing to the retrospective design and deidentification of the data, patient consent was not needed for this study.

### Inclusion criteria

The age range of the patients with CP was from 0 to 18 years. The cases of repeated admission were based on the medical records from the last hospitalization. The medical records had to include detailed primary, perinatal, maternal, and pregnancy data. From these records, 9 major risk factors in children with CP were identified. They were:

- Preterm birth or low birth weight (gestational age < 37 weeks, weight < 2500 g);
- Comorbidities or risk factors during pregnancy (such as upper respiratory tract infection, intrauterine infection, threatened abortion, hypertension, diabetes, nephritis, renal cyst, hepatitis, hyperthyroidism or hypothyroidism, cytomegalovirus infection, threatened abortion, vaginal bleeding, long-term exposure to industrial chemical reagents, smoking, poor medication adherence, intellectual disability, anaemia, malnutrition and maternal age > 34 years);
- Asphyxia;
- hypoxic-ischaemic encephalopathy (HIE);
- Placental abnormalities (placental dysplasia, ageing, previa or abruption), umbilical cord abnormalities (umbilical cord around the neck, torsion, a cord that is too short or too thin), amniotic fluid abnormalities (amniotic fluid turbidity, premature rupture, and too little fluid);
- Hyperbilirubinemia;
- Multiple gestation;
- Traumatic brain injury/intracranial haemorrhage;
- Neonatal seizure.

### Diagnostic criteria for CP

The diagnostic criteria for CP had to adhere to the 2015 Guidelines for CP in China [10]. These criteria include (a) persistent central dyskinesia, (b) abnormal movement and posture development, (c) abnormal muscle tension and strength, and (d) abnormal reflex development. The guidelines reference the 2006 edition of the International Standard for the Definition, Classification, and Grading of CP [1].

### Classification and GMFCS of CP

In the 2015 Chinese guidelines for CP, the classification of CP included spastic diplegia, spastic hemiplegia, spastic quadriplegia, dyskinesia, ataxia, and the mixed type. The Gross Motor Function Classification System (GMFCS) includes grades I-V, with grades I and II indicating the ability to walk, grade III indicating the use of mobile devices, and grades IV and V indicating the need for a wheelchair for mobility. Generally, as the GMFCS level increases, the severity of CP also increases.

### Diagnostic criteria of comorbidity

Diagnostic criteria for comorbidities:

- Epilepsy: The diagnosis of epilepsy was based on the criteria established by the International League Against Epilepsy (ILAE) in 2014. These criteria include the following:
  1. Experiencing at least two unprovoked (or reflex) seizures with an interval of more than 24 h between them.
  2. Experiencing one unprovoked (or reflex) seizure and having a likelihood of further seizures similar to the general recurrence risk (at least 60%) within the next ten years.
  3. Diagnosis of an epilepsy syndrome.
- Intellectual disability: The diagnostic criteria are as follows:
  1. For children under three years of age, the Gesell Development Scale was used, with adaptability as an indicator of intelligence.
  2. For children aged 4–6 years, the Wechsler Intelligence Scale for Children (WPPSI) was employed.
  3. For children aged 6–16 years, the Wechsler Intelligence Scale for Children (WISC-R) was used. The severity levels of intellectual disability were categorized as mild (IQ = 50–69), moderate (IQ = 35–49), or severe (IQ < 35).

Hearing impairment: The diagnostic criteria for hearing impairment were based on brainstem auditory evoked potential (BAEP) tests. Specifically, hearing impairment was confirmed when the V wave was not elicited, using the standard of more than 30 dB nHL (decibel normalized hearing level).

### Statistical analysis

This study was divided into three parts. Part I: Fig. 2 was produced in R v3.6.2. (R Foundation for Statistical Computing, Vienna, Austria) via the R package UpSetR v. 1.4.0 (https://cran.r-project.org/web/packages/UpSetR/index.html). This figure reflects the quantitative characteristics of risk factors in children with CP. In addition, we also conducted a descriptive analysis of the characteristics of children with CP under different individual risk factors. We used the likelihood ratio χ2 test to compare the intergroup characteristics of children with CP under different single risk factors. The count data are expressed as [n(%)], and *P* < 0.05 was considered statistically significant. Part II: Univariate logistic regression was used to analyse the influencing factors affecting the characteristics of CP classification, the GMFCS level and comorbidities, and multivariate logistic regression was used to adjust for confounding factors. In Part III, a proportional histogram was used to describe the number of risk factors in the classification, GMFCS level and comorbidities of children with CP, and univariate logistic regression was used to analyse the influencing factors affecting the classification, GMFCS level and comorbidities. Statistical analysis was performed via SPSS v25 (IBM Corp., Armonk, NY, USA).

## Results

### Clinical characteristics of children with CP

**Fig. 1:**
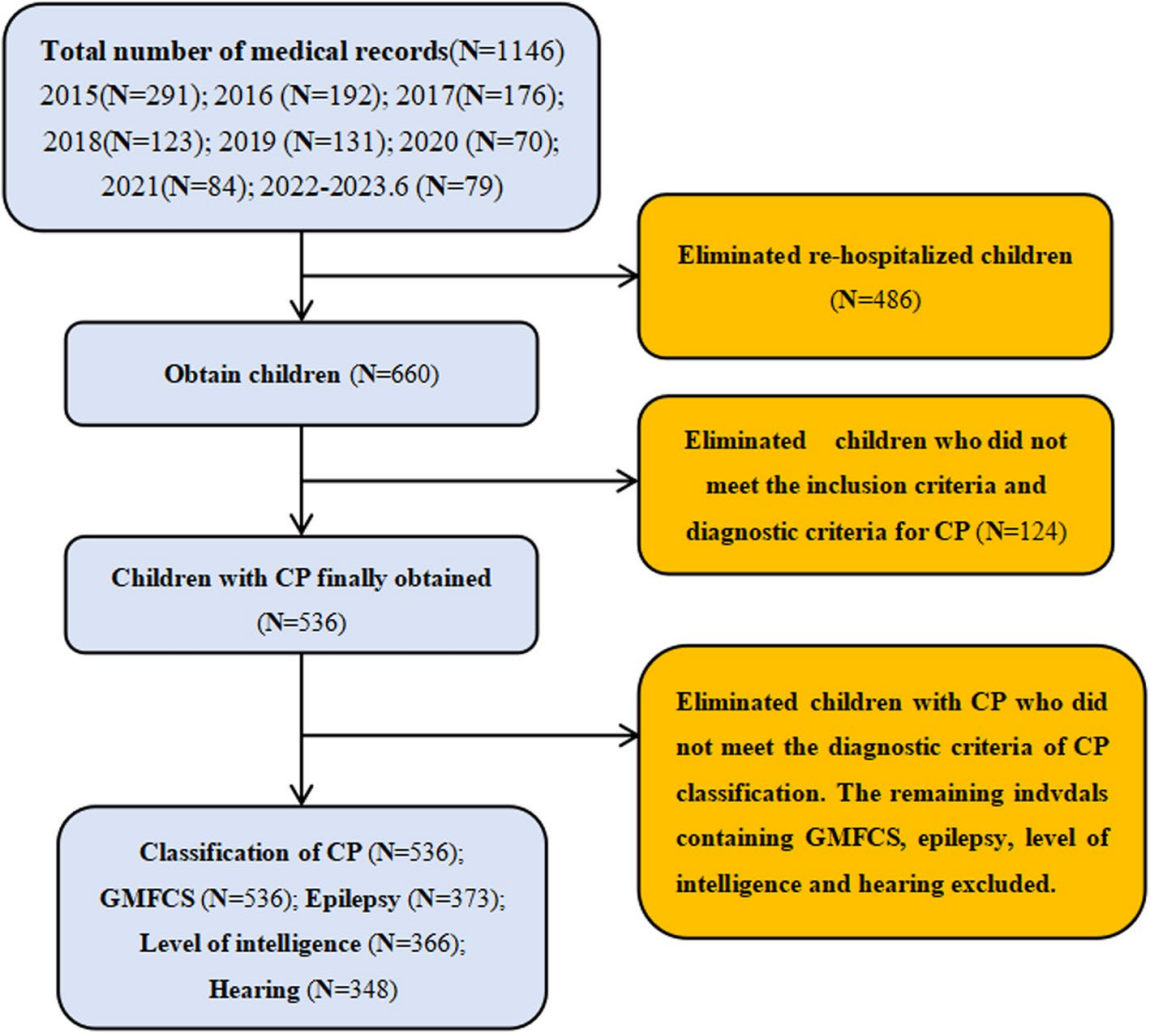
Flow chart of participant screening

Among the 1146 children whose medical records were reviewed from January 2015 to June 2023, 536 children with CP met the inclusion criteria after children with repeated admissions and individuals who did not meet the diagnostic criteria for CP classification were excluded. As indicated in Fig. 1, among these 536 CP patients, 243 (45.3%) were born prematurely or had low birth weights, 212 (39.6%) had comorbidities and/or risk factors during gestation, 152 (28.3%) experienced asphyxia, 151 (28.2%) had HIE, 142 (26.5%) had hyperbilirubinemia, 46 (8.6%) were from multiple gestations, 45 (8.4%) had experienced brain trauma or intracranial haemorrhage, and 43 (8.0%) experienced neonatal seizures.

**Fig. 2:**
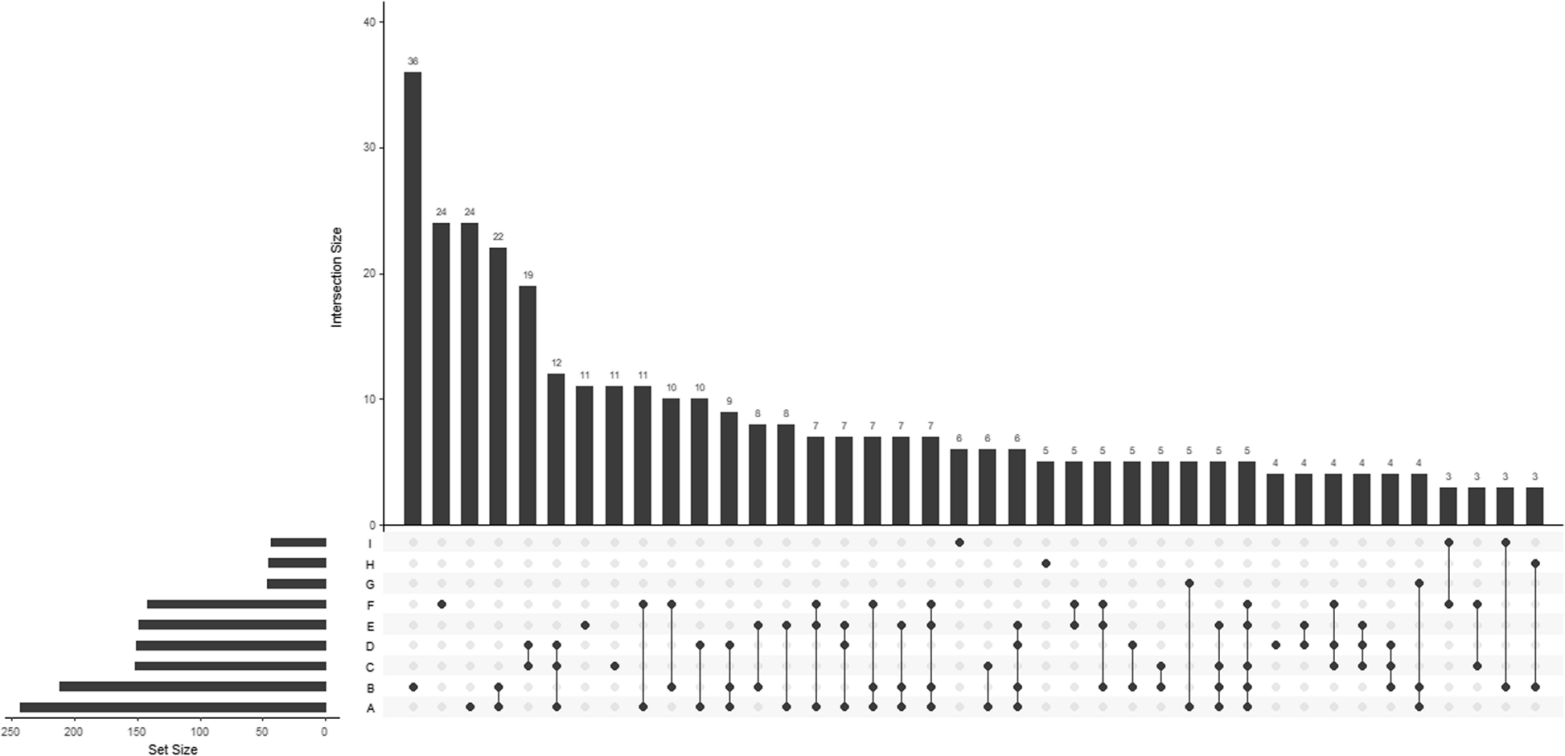
UpSet diagram of risk factors for CP. Note: A: Premature birth and/or low birth weight; B: High-risk factors and/or maternal comorbidities during pregnancy; C: Asphyxia; D: HIE; E: Placental/umbilical cord/ amniotic fluid abnormalities; F: Hyperbilirubinemia; G: Multiparous; H: Intracranial haemorrhage; I: Neonatal seizures. In the top-panel bar chart, the first bar represents 36 individuals with only Factor B (high-risk factors and/or maternal comorbidities during pregnancy), and the fourth bar represents individuals with only Factors A and B. The highest bar in the bottom panel, located in the left corner, indicates approximately 243 individuals with Factor A (premature birth and/or low birth weight)

Among the children with CP and a single risk factor, the highest prevalence (6.7%) was observed for those with comorbidities or risk factors during gestation. For children with CP and two risk factors, the highest prevalence (4.1%) was found in those with preterm birth or low birth weight and pregnancy-related risk factors or comorbidities. Among children with CP and three risk factors, the highest prevalence (2.2%) was noted for those with a combination of preterm birth or low birth weight, asphyxia, and HIE. Children with CP and four risk factors, including preterm birth or low birth weight, pregnancy-related risk factors or comorbidities, umbilical cord, placental, or amniotic fluid abnormalities, and hyperbilirubinemia exhibited the highest prevalence (1.3%) (Fig. 2).

The table presents the characteristics of children with CP under different risk factors. The χ^2^ test revealed that risk factors had no significant association with sex or the method of birth (*P* > 0.05). The number of individuals treated between 2019.01 and 2023.06 decreased to half that treated between 2015 and 2018, likely because the population shifted to southern China because of better economic development. This led to a significant decrease in the population in the northeast region. Additionally, due to COVID-19, many parents may have refused to visit the hospital for rehabilitation treatment to avoid contracting the virus. In terms of seasons, the risk factors and comorbidities during pregnancy in the relatively cold spring and winter in Northeast China were significantly greater than those in the other two, relatively warm seasons (*P* = 0.028), possibly because cold weather may increase the likelihood of adverse risk factors and comorbidities during pregnancy. Furthermore, the number of risk factors and comorbidities during pregnancy, as well as multiple gestations, may increase the risk of preterm birth and low birth weight (*P* < 0.001). In terms of classification, most risk factors were more common in patients with spastic quadriplegia and mixed types than in patients with other types. Preterm birth and low birth weight were more common in patients with spastic diplegia, whereas risk factors and comorbidities during pregnancy were more common. Hyperbilirubinemia was more common in patients with the dyskinetic type. Concerning the GMFCS level, we observed that the incidence of asphyxia gradually increased with increasing grade (*P* = 0.019) (Table 1).

**Table 1:** Characteristics of children with CP with different individual risk factors (n(%))

	Total	Preterm birth and/or low birth weight	Pregnancy risks and/or comorbidities	Asphyxia	HIE	Placental/umbilical cord/amniotic fluid abnormalities	Hyperbilirubinemia	Multiparous	Traumatic brain injury/intracranial haemorrhage	Neonatal seizures
Year(Mean ± SD)	4.83 ± 3.45	5.12 ± 3.61	4.24 ± 2.98	5.15 ± 3.95	4.92 ± 3.47	5.02 ± 3.55	4.46 ± 3.10	4.41 ± 3.23	4.84 ± 4.80	4.91 ± 3.47
Sex										
Male	360	167 (46.4)	140 (38.9)	99 (27.5)	96 (26.7)	101 (28.1)	101 (28.1)	28 (7.8)	30 (8.3)	27 (7.5)
Female	176	76 (43.2)	72 (40.9)	53 (30.1)	55 (31.3)	48 (27.3)	41 (23.3)	18 (10.2)	15 (8.5)	16 (9.1)
Year of collection										
2015–2018	358	171 (47.8)	134 (37.4)	99 (27.7)	104 (29.1)	92 (25.7)	85 (23.7)	35 (9.8)	33 (9.2)	26 (7.3)
2019–2023	178	72 (40.4)	78 (43.8)	53 (29.8)	47 (26.4)	57 (32.0)	57 (32.0)	11 (6.2)	12 (6.7)	17 (9.6)
Season										
Spring	121	56 (46.3)	55 (45.5)	37 (30.6)	29 (24.0)	36 (29.8)	32 (26.4)	12 (9.9)	9 (7.4)	10 (8.3)
Summer	180	77 (42.8)	71 (39.4)	51 (28.3)	53 (29.4)	48 (26.7)	56 (31.1)	16 (8.9)	14 (7.8)	16 (8.9)
Autumn	108	52 (48.1)	30 (27.8)	34 (31.5)	36 (33.3)	29 (26.9)	24 (22.2)	13 (12.0)	7 (6.5)	8 (7.4)
Winter	127	58 (45.7)	56 (44.1)	30 (23.6)	33 (26.0)	36 (28.3)	30 (23.6)	5 (3.9)	15 (11.8)	9 (7.1)
Gestational age (w)										
≥ 37	313	20 (6.4)	102 (32.6)	88 (28.1)	76 (24.3)	69 (22.0)	70 (22.4)	10 (3.2)	22 (7.0)	32 (10.2)
32 to 36	127	127 (100)	64 (50.4)	37 (29.1)	43 (33.9)	48 (37.8)	48 (37.8)	15 (11.8)	12 (9.4)	8 (6.3)
28 to 31	82	82 (100)	37 (45.1)	19 (23.2)	27 (32.9)	28 (34.1)	16 (19.5)	18 (22.0)	10 (12.2)	2 (2.4)
< 28	14	14 (100)	9 (64.3)	8 (57.1)	5 (35.7)	4 (28.6)	8 (57.1)	3 (21.4)	1 (7.1)	1 (7.1)
Birth weight (g)										
≥ 2500	359	66 (18.4)	129 (35.9)	98 (27.3)	91 (25.3)	82 (22.8)	94 (26.2)	12 (3.3)	27 (7.5)	35 (9.7)
1500 to 2499	134	134 (100)	61 (45.5)	41 (30.6)	42 (31.3)	52 (38.8)	36 (26.9)	20 (14.9)	13 (9.7)	7 (5.2)
1000 to 1499	40	40 (100)	19 (47.5)	11 (27.5)	17 (42.5)	15 (37.5)	11 (37.5)	12 (30.0)	5 (12.5)	1 (2.5)
< 1000	3	3 (100)	3 (100)	2 (66.7)	1 (33.3)	0 (0)	1 (33.3)	2 (66.7)	0 (0)	0 (0)
Method of delivery										
Eutocia	238	115 (48.3)	100 (42.0)	67 (28.2)	72 (30.3)	67 (28.2)	62 (26.1)	20 (8.4)	15 (6.3)	22 (9.2)
Caesarean section	298	128 (43.0)	112 (37.6)	85 (28.5)	79 (26.5)	82 (27.5)	80 (26.8)	26 (8.7)	30 (10.1)	21 (7.0)
Classification of CP										
Spastic diplegia	211	130 (61.6)	91 (43.1)	56 (26.5)	67 (31.8)	63 (29.9)	55 (26.1)	24 (11.4)	18 (8.5)	12 (5.7)
Spastic hemiplegia	126	36 (28.6)	53 (42.1)	24 (19.0)	20 (15.9)	27 (21.4)	19 (15.1)	6 (4.8)	11 (8.7)	8 (6.3)
Spastic quadriplegia	74	37 (50.0)	30 (40.5)	27 (36.5)	28 (37.8)	30 (40.5)	14 (18.9)	10 (13.5)	7 (9.5)	8 (10.8)
Dyskinesia	86	28 (32.6)	25 (29.1)	23 (26.7)	21 (24.4)	19 (22.1)	44 (51.2)	5 (5.8)	7 (8.1)	8 (9.3)
Ataxia	9	3 (33.3)	3 (33.3)	2 (22.2)	1 (11.1)	1 (11.1)	2 (22.2)	0 (0)	0 (0)	0 (0)
Mixed	30	9 (30.0)	10 (33.3)	20 (66.7)	14 (46.7)	9 (30.0)	8 (26.7)	1 (3.3)	2 (6.7)	7 (23.3)
GMFCS level										
I	170	72 (42.4)	79 (46.5)	35 (20.6)	44 (25.9)	49 (28.8)	38 (22.4)	11 (6.5)	16 (9.4)	9 (5.3)
II	100	45 (45.0)	32 (32.0)	27 (27.0)	19 (19.0)	23 (23.0)	32 (32.0)	7 (7)	7 (7.0)	8 (8.0)
III	97	53 (54.6)	40 (41.2)	28 (28.9)	34 (35.1)	26 (26.8)	26 (26.8)	10 (10.3)	3 (3.1)	5 (5.2)
IV	89	39 (43.8)	37 (41.6)	30 (33.7)	25 (28.1)	31 (34.8)	25 (28.1)	7 (7.9)	6 (6.7)	11 (12.4)
V	80	34 (42.5)	24 (30.0)	32 (40.0)	29 (36.3)	20 (25.0)	21 (26.3)	11 (13.8)	13 (16.3)	10 (12.5)
Epilepsy										
No	277	127 (45.8)	120 (43.3)	76 (27.4)	87 (31.4)	71 (25.6)	76 (27.4)	28 (10.1)	27 (9.7)	14 (5.1)
Epileptiform discharges	57	25 (43.9)	21 (36.8)	24 (42.1)	18 (31.6)	18 (31.6)	16 (28.1)	7 (12.3)	3 (5.3)	5 (8.8)
Seizures	39	17 (43.6)	17 (43.6)	10 (25.6)	8 (20.5)	9 (23.1)	7 (17.9)	2 (5.1)	2 (5.1)	13 (33.3)
Level of Intelligence										
Normal	123	52 (43.2)	50 (40.7)	27 (22.0)	25 (20.3)	30 (24.4)	22 (17.9)	5 (4.1)	10 (8.1)	4 (3.3)
Mild disability	104	46 (44.2)	48 (46.2)	28 (26.9)	29 (27.9)	26 (25.0)	30 (28.8)	11 (10.6)	5 (4.8)	9 (8.7)
Moderate disability	64	36 (56.3)	22 (34.4)	21 (32.8)	18 (28.1)	19 (29.7)	23 (35.9)	5 (7.8)	9 (14.1)	5 (7.8)
Severe disability	75	24 (32.0)	29 (38.7)	25 (33.3)	26 (34.7)	24 (32.0)	29 (38.7)	7 (9.3)	5 (6.7)	8 (10.7)
Hearing										
Normal	289	142 (49.1)	123 (42.6)	82 (28.4)	92 (31.8)	68 (23.5)	73 (25.3)	19 (6.6)	22 (7.6)	11 (3.8)
Abnormal	59	18 (30.5)	25 (42.4)	16 (27.1)	16 (27.1)	23 (39.0)	21 (35.6)	6 (10.2)	5 (8.5)	11 (18.6)

### Effects of individual risk factors on classification, the GMFCS level and comorbidities in children with CP

Logistic regression analysis (Table 2) revealed that asphyxia was positively correlated with epileptic discharges (adjusted odds ratio [OR] = 2.108 [1.11 ~ 4.001], *P* = 0.023), whereas neonatal seizures were significantly associated with epilepsy (adjusted OR = 12.402 [4.83–31.848], *P* < 0.001). Ordered logistic regression analysis was employed for assessing intelligence levels, with the results indicating significant correlations. Specifically, HIE (adjusted OR = 1.791 [1.130–2.838], *P* = 0.013) and hyperbilirubinemia (adjusted OR = 2.328 [1.516–3.575], *P* < 0.001) were found to be linked to variations in intelligence levels. With respect to hearing impairment, children with CP born prematurely presented a reduced likelihood of hearing disabilities (adjusted OR = 0.372 [0.1840.75], *P* = 0.006). Conversely, the presence of umbilical cord/placenta/amniotic fluid abnormalities (adjusted OR = 2.314 [1.224.389], *P* = 0.01) and neonatal seizures (adjusted OR = 5.206 [1.98 ~ 13.683], *P* = 0.001) were positively associated with hearing disabilities.

**Table 2:** Univariate or multivariate logistic regression analysis of the effect of single risk factors on the comorbidity, GMFCS and classification of CP

	**Epileptiform Discharges vs. None**		**Seizures vs. None**		**Level of Intelligence (ordered)**		**Hearing impairment vs. None**		**GMFCS levels IV and V vs. levels I, II and III**	
	**OR (95% CI)**	**P**	**OR (95% CI)**	**P**	**OR (95% CI)**	**P**	**OR (95% CI)**	**P**	**OR (95% CI)**	**P**
Preterm birth and/or low birth weight vs. None (Reference)	0.923(0.52~1.638)	0.784	0.913(0.464~1.794)	0.791	0.914(0.630~1.327)	0.637	0.454(0.249~0.828)	0.01	0.881(0.61~1.272)	0.499
							0.372(0.184~0.75)*	0.006		
Pregnancy risks and/or comorbidities vs. None (Reference)	0.763(0.424~1.374)	0.368	1.011(0.514~1.988)	0.975	0.893(0.613~1.300)	0.554	0.992(0.563~1.749)	0.979	0.808(0.554~1.177)	0.267
Asphyxia vs. None (Reference)	1.923(1.068~3.464)	0.029	0.912(0.424~1.961)	0.814	1.517(1.004~2.293)	0.048	0.939(0.501~1.761)	0.845	1.783(1.204~2.642)	0.004
	2.108(1.11~4.001)*	0.023			1.241(0.796~1.935)*	0.34			1.619(1.052~2.49)*	0.028
HIE vs. None (Reference)	1.008(0.546~1.861)	0.98	0.564(0.249~1.277)	0.169	1.589(1.047~2.411)	0.03	0.797(0.426~1.489)	0.476	1.307(0.878~1.946)	0.187
					1.791(1.130~2.838)*	0.013				
Placenta/umbilical cord/amniotic fluid abnormalities vs. None (Reference)	1.339(0.720~2.490)	0.356	0.870(0.394~1.922)	0.731	1.307(0.863~1.978)	0.206	2.076(1.151~3.744)	0.015	1.186(0.794~1.773)	0.404
							2.314(1.22~4.389)*	0.01		
Hyperbilirubinemia vs. None (Reference)	1.032(0.547~1.948)	0.922	0.579(0.245~1.366)	0.212	2.059(1.363~3.111)	0.001	1.635(0.902~2.966)	0.105	1.056(0.7~1.592)	0.796
					2.328(1.516~3.575)*	<0.001				
Multiparous vs. None (Reference)	1.245(0.515~3.008)	0.626	0.481(0.11~2.102)	0.331	1.564(0.783~3.121)	0.206	1.609(0.614~4.218)	0.334	1.443(0.775~2.689)	0.248
Traumatic brain injury/intracranial haemorrhage Vs None (Reference)	0.514(0.151~1.757)	0.289	0.501(0.114~2.193)	0.358	1.110(0.561~2.192)	0.766	1.124(0.408~3.098)	0.822	1.661(0.892~3.094)	0.11
Neonatal seizures vs. None (Reference)	1.806(0.624~5.232)	0.276	9.393(3.992~22.102)	<0.001	1.990(0.970~4.084)	0.061	5.792(2.378~14.105)	<0.001	2.225(1.187~4.17)	0.013
			12.402(4.83~31.848)*	<0.001			5.206(1.98~13.683)*	0.001	1.998(1.048~3.811)*	0.036

	**Spastic Hemiplegia vs. Spastic Diplegia**		**Spastic Quadriplegia vs. Spastic Diplegia**		**Dyskinesia vs. Spastic Diplegia**		**Ataxia vs. Spastic Diplegia**		**Mixed vs. Spastic Diplegia**	
	**OR (95% CI)**	**P**	**OR (95% CI)**	**P**	**OR (95% CI)**	**P**	**OR (95% CI)**	**P**	**OR (95% CI)**	**P**
Preterm birth and/or low birth weight vs. None (Reference)	0.249(0.155~0.401)	<0.001	0.623(0.365~1.062)	0.082	0.301(0.177~0.511)	<0.001	0.312(0.076~1.28)	0.106	0.267(0.117~0.612)	0.002
	0.293(0.176~0.485)*	<0.001			0.298(0.167~0.531)*	<0.001			0.315(0.128~0.777)*	0.012
Pregnancy risks and/or comorbidities vs. None (Reference)	0.957(0.613~1.496)	0.848	0.899(0.525~1.54)	0.698	0.54(0.315~0.927)	0.025	0.659(0.161~2.707)	0.563	0.659(0.294~1.477)	0.311
					0.671(0.378~1.193)*	0.175				
Asphyxia vs. None (Reference)	0.651(0.38~1.117)	0.119	1.59(0.905~2.793)	0.107	1.01(0.573~1.781)	0.971	0.791(0.16~3.92)	0.774	5.536(2.442~12.547)	<0.001
									5.429(2.184~13.494)*	<0.001
HIE vs. None (Reference)	0.406(0.232~0.709)	0.002	1.308(0.753~2.272)	0.34	0.694(0.392~1.229)	0.211	0.269(0.033~2.192)	0.22	1.881(0.868~4.076)	0.11
	0.46(0.248~0.851)*	0.013								
Placenta/umbilical cord/amniotic fluid abnormalities vs. None (Reference)	0.641(0.382~1.075)	0.092	1.602(0.924~2.776)	0.093	0.666(0.37~1.2)	0.176	0.294(0.036~2.397)	0.253	1.007(0.437~2.32)	0.987
Hyperbilirubinemia vs. None (Reference)	0.504(0.283~0.897)	0.02	0.662(0.343~1.278)	0.219	2.971(1.762~5.012)	<0.001	0.81(0.163~4.019)	0.797	1.031(0.434~2.451)	0.944
	0.551(0.3~1.013)*	0.055			3.589(2.037~6.322)*	<0.001				
Multiparous vs. None (Reference)	0.39(0.155~0.981)	0.045	1.217(0.552~2.684)	0.626	0.481(0.177~1.305)	0.151			0.269(0.035~2.063)	0.206
	0.621(0.237~1.633)*	0.334								
Traumatic brain injury/intracranial haemorrhage Vs None (Reference)	1.026(0.468~2.248)	0.95	1.12(0.448~2.8)	0.808	0.95(0.382~2.364)	0.912			0.766(0.169~3.48)	0.73
Neonatal seizures vs. None (Reference)	1.124(0.447~2.83)	0.804	2.01(0.788~5.13)	0.144	1.701(0.67~4.32)	0.264			5.047(1.807~14.098)	0.002
									3.703(1.237~11.082)*	0.019

Note: *Adjusted OR. Adjusted by preterm birth and/or low birth weight, pregnancy risks and/or comorbidities, asphyxia, HIE, placental/umbilical cord/amniotic fluid abnormalities, hyperbilirubinemia, multiparity, traumatic brain injury/intracranial haemorrhage, and neonatal seizures

Given that the GMFCS level did not meet the requirements for ordered logistic regression analysis, we opted for binary logistic regression analysis. We categorized the GMFCS level into two groups: Levels I to III (representing mild to moderate) and Levels IV and V (indicating severe impairment). In this analysis, asphyxia (adjusted OR = 1.619 [1.052–2.49], *P* = 0.028) and neonatal seizures (adjusted OR = 1.998 [1.048–3.811], *P* = 0.036) were found to be associated with higher GMFCS levels.

Risk factors and/or comorbidities during pregnancy were significantly associated with preterm birth and/or low birth weight. In patients with spastic diplegia, the ORs for preterm birth and/or low birth weight and related pregnancy risk factors and comorbidities were consistently less than 1. These findings suggest that preterm birth and/or low birth weight are significant risk factors for patients with spastic diplegia, with a more pronounced effect in patients with spastic hemiplegia and dyskinetic types (adjusted OR < 0.5, *P* < 0.01).

Compared with patients with spastic diplegia, hyperbilirubinemia was more prevalent in patients with the dyskinetic type (adjusted OR = 3.589 [2.037 ~ 6.322], *P* < 0.001), whereas patients with mixed-type CP had a higher likelihood of experiencing asphyxia (adjusted OR = 5.429 [2.184 ~ 13.494], *P* < 0.001) and neonatal seizures (adjusted OR = 3.703 [1.237 ~ 11.082], *P* = 0.019). The possibility of ataxia occurring in conjunction with risk factors was lower (Crude OR < 1).

### Effects of the number of risk factors on the classification, GMFCS level and comorbidities of children with CP

The distribution of the number of risk factors was as follows: 60 patients (11.2%) had no risk factors, 123 patients (22.9%) had one risk factor, 147 patients (27.4%) had two risk factors, 109 patients (20.3%) had three risk factors, 58 patients (10.8%) had four risk factors, 28 patients (5.2%) had five risk factors, and 10 patients (1.9%) had six risk factors. One child with CP had a combination of seven risk factors, but for ease of analysis, we included this child in the six-risk factor group.

**Fig. 3:**
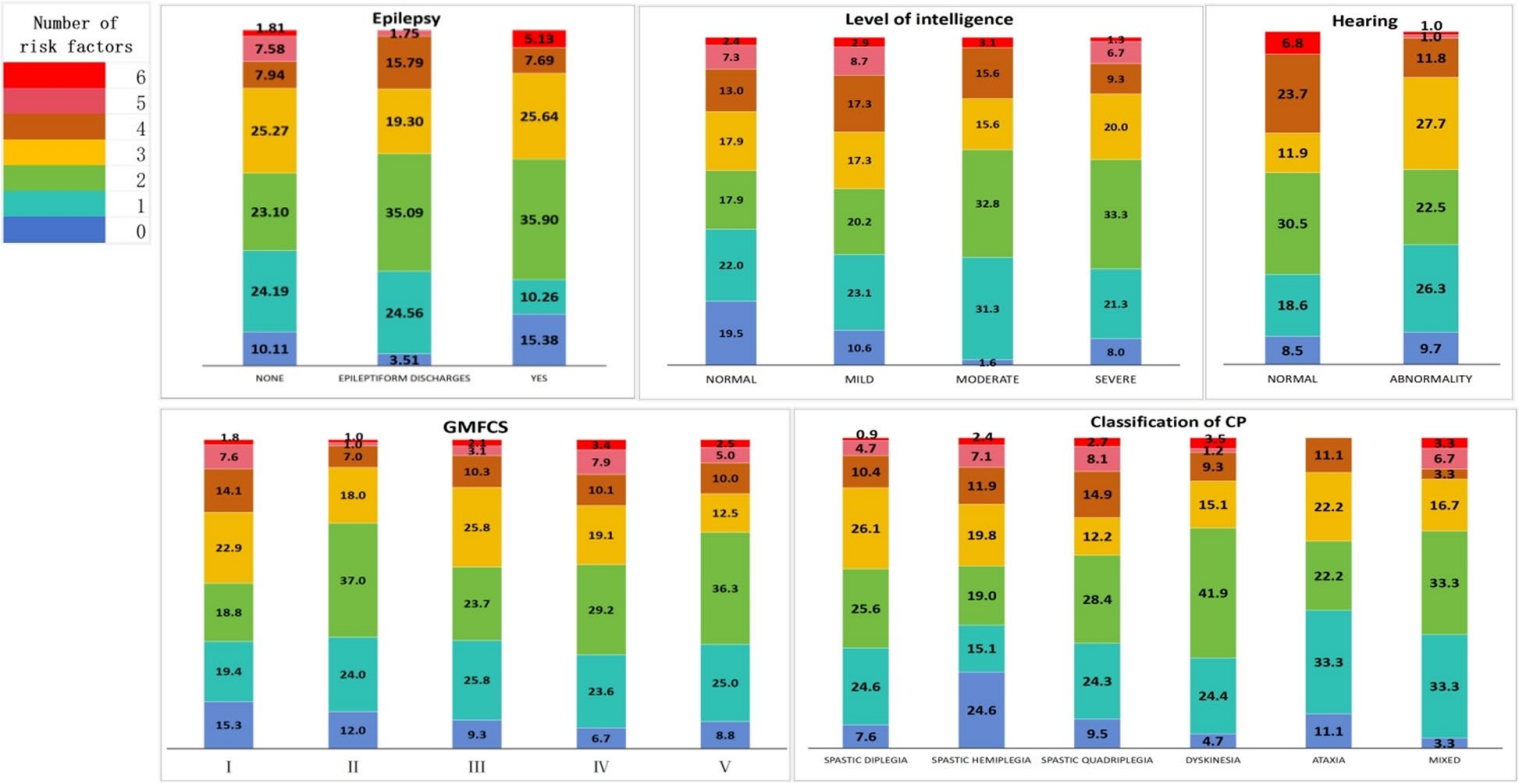
The characteristics of the number of risk factors according to CP classification, GMFCS level, intellectual disability, epilepsy and hearing impairment

As shown in Fig. 3, our observations revealed that in terms of comorbidities, the majority of children with CP who had epilepsy and hearing impairment appeared to be more concentrated within the group with ≤ 3 risk factors. When considering the GMFCS, Grade I had the highest incidence of risk factors ≥ 4, followed by Grades IV and V. In the CP classification, children with spastic quadriplegia accounted for the highest proportion of cases with ≥ 4 risk factors, whereas those with spastic hemiplegia accounted for the highest proportion of cases with no identified risk factors.

For logistic regression analysis (Table 3), we used ordinal logistic regression analysis to analyse the level of intelligence (parallel line test *P* = 0.114 > 0.05, model fitting information = 0.0051). Nevertheless, the difference was statistically significant for only the combination of 1‒4 risk factors (*P* < 0.05). We used ordered logistic regression analysis for the GMFCS level (parallel line test *P* = 0.21 > 0.05, model fitting information = 0.0381), but the difference was significant only for the presence of one or two risk factors (*P* < 0.05). Compared with spastic diplegia, spastic hemiplegia and ataxia CP were less common in children with various risk factors (crude OR < 1), spastic hemiplegia was more statistically significant in those with 1–4 risk factors (*P* < 0.05), spastic quadriplegia was more common in those with ≥ 4 risk factors (crude OR > 1), and the mixed type was more common in those with most risk factors (crude OR > 1, excluding 4 risk factors).

**Table 3:** Logistic regression analysis of the effect of multiple risk factors on the comorbidities, GMFCS level and classification of CP

Number of risk factors	Epileptiform Discharges vs. None		Seizures vs. None		Level of Intelligence (ordered)		Hearing impairment vs. None		GMFCS(ordered)	
	OR（95% CI）	*P*	OR（95% CI）	*P*	OR（95% CI）	*P*	OR（95% CI）	*P*	OR（95% CI）	*P*
0 (Reference)	1		1		1		1		1	
1	2.925(0.623~13.726)	0.174	0.279(0.073~1.064)	0.062	2.883(1.432~5.807)	0.003	0.811(0.259~2.541)	0.719	1.923(1.101~3.364)	0.022
2	4.375(0.957~20.001)	0.057	1.021(0.356~2.93)	0.969	4.345(2.155~8.767)	<0.001	1.551(0.524~4.591)	0.428	2.232(1.296~3.850)	0.004
3	2.2(0.458~10.565)	0.325	0.667(0.221~2.009)	0.471	2.765(1.327~5.755)	0.007	0.49(0.144~1.669)	0.254	1.330(0.751~2.351)	0.328
4	5.727(1.121~29.253)	0.036	0.636(0.143~2.835)	0.553	2.408(1.116~5.197)	0.025	2.306(0.74~7.189)	0.15	1.292(0.673~2.479)	0.442
5	0.667(0.057~7.852)	0.747	/	/	1.866(0.723~4.821)	0.198	/	/	1.379(0.615~3.089)	0.436
6	/	/	1.867(0.29~12.013)	0.511	2.252(0.601~8.440)	0.228	7.467(1.267~44.002)	0.026	2.537(0.808~7.957)	0.111

	Spastic Hemiplegia vs. Spastic Diplegia		Spastic Quadriplegia vs. Spastic Diplegia		Dyskinesia vs. Spastic Diplegia		Ataxia vs. Spastic Diplegia		Mixed vs. Spastic Diplegia	
Number of risk factors		*P*		*P*		*P*		*P*		*P*
0 (Reference)	1		1		1		1		1	
1	0.189(0.085~0.420)	<0.001	0.791(0.28~2.233)	0.658	1.615(0.483~5.402)	0.436	0.923(0.090~9.502)	0.946	3.077(0.365~25.908)	0.301
2	0.465(0.106~0.496)	<0.001	0.889(0.32~2.468)	0.821	2.667(0.824~8.627)	0.102	0.593(0.050~6.967)	0.677	2.963(0.352~24.933)	0.318
3	0.235(0.109~0.505)	<0.001	0.374(0.12~1.162)	0.089	0.945(0.271~3.304)	0.930	0.582(0.050~6.839)	0.667	1.455(0.158~13.366)	0.741
4	0.352(0.144~0.858)	0.022	1.143(0.363~3.594)	0.819	1.455(0.373~5.679)	0.590	0.727(0.042~12.518)	0.826	0.727(0.042~12.518)	0.826
5	0.465(0.157~1.373)	0.166	1.371(0.357~5.272)	0.646	0.400(0.039~4.109)	0.441	/	/	3.200(0.256~40.056)	0.367
6	0.774(0.117~5.115)	0.790	2.286(0.266~19.658)	0.451	6.000(0.736~48.9)	0.094	/	/	8.000(0.347~184.364)	0.194

## Discussion

### Clinical characteristics of CP

#### Prenatal risk factors

The causes of CP in children are multifaceted. According to epidemiological studies, 70% to 80% of CP cases are associated with prenatal factors such as comorbidities or risk factors during pregnancy; abnormalities in the placenta, umbilical cord, or amniotic fluid; and multiple gestation [11]. In our research, we observed that 39.6% of children with CP had one or more risk factors during gestation. The highest number of children (6.7%) with CP had only one risk factor, and preterm birth and low birth weight were common risk factors in children with spastic diplegia. Additionally, our study revealed that the occurrence of comorbidities or risk factors during gestation was significantly greater in the colder seasons (*P* = 0.028) in Northeast China, possibly because of increased viral infections, particularly influenza, during the winter. Importantly, pregnant women are particularly vulnerable to infections during the second trimester [12]. Furthermore, a study conducted in a region similar to Jiamusi, California, reported a slight increase in CP risk among children conceived in winter and spring, suggesting a potential association between seasonal variations in environmental factors and CP aetiology [13]. Griffiths et al. [14] reported that when a stillbirth occurs in twin pregnancies, the incidence of CP in the surviving foetus increases by approximately 11%, and amniotic fluid contamination is also a high-risk factor leading to CP. Karimzadeh et al. [14] analysed the influence of II–III degree amniotic fluid contamination on the incidence of neonatal HIE through case‒control studies. The results showed that amniotic fluid contamination of the II and III degree increase the incidence of HIE in CP patients. Comprehensive monitoring of various risk factors and timely intervention can significantly reduce the risk of HIE.

#### Risk factors during birth

Neonatal asphyxia during delivery and premature delivery are high-risk factors for CP. Epidemiological investigations have shown that preterm infants account for approximately 7% of all surviving newborns, but CP in preterm newborns accounts for approximately 40% of all CP cases [15].

A previous study revealed a significant correlation between preterm birth and CP in children [16]. In our study, 243 individuals (45.3%) were born premature or had a low birth weight, which are the most common risk factors for spastic diplegia. Preterm delivery increases the risk of CP by 7.11 times because the organs of premature infants are still immature. Their ability to tolerate hypoxia is poor, which can cause developmental defects in the nervous system, especially in the periventricular white matter area, leading to periventricular white matter damage [17].

Neonatal asphyxia often occurs as a result of distress during childbirth. Asphyxiating brain injury is caused by events such as reduced blood flow, a lack of oxygen, and the restoration of blood flow after resuscitation [18]. The underlying mechanisms involve oxidative stress from inflammatory reactions, the formation of free radicals, and the death of vulnerable areas in the brain, leading to injury, particularly in premature infants.

#### Risk factors after birth

When neonatal hyperbilirubinemia occurs, free bilirubin can pass through the blood‒brain barrier and combine with brain cells, causing neurotoxicity and resulting in brain cell damage. The site of central nervous system injury caused by bilirubin is located mainly in the extrapyramidal system, which is more common in patients with dyskinetic CP [19]. A study revealed that 142 cases (26.5%) of hyperbilirubinemia were found in patients with dyskinetic CP, which is consistent with the findings of Saini et al.’s report [20]. In premature infants at high risk for CP, neonatal intracranial haemorrhage is a primary neurological comorbidity. The pathophysiological mechanisms include the inherent fragility of germinal stromal blood vessels, fluctuations in cerebral blood flow, and coagulation disorders, all of which contribute to brain injury in premature infants [21]. According to studies by Mert et al. [22], children with CP who have experienced neonatal seizures are 3.3 times more likely to have a poor prognosis than are those without a history of neonatal seizures. This may be due to the negative impact of neonatal seizures on neurodevelopment, leading to brain lesions that can contribute to cognitive, behavioural, or seizure issues later in life [23].

### Effects of individual risk factors on CP

#### Classification

The occurrence of spastic diplegia is more likely in babies who are born prematurely or have low birth weight. These findings suggest that premature birth significantly increases the risk of spastic diplegia. Sukhov et al. [24] highlighted the significant impact of premature birth on the development of CP. Furthermore, Hirvonen et al. [25] reported that the incidence of CP in children decreases nonlinearly with increasing gestational age.

Additionally, asphyxia (OR = 5.429) and neonatal seizures (OR = 3.70) were more common in patients with mixed CP. This may be because these risk factors often lead to injury across multiple brain regions, such as the cerebral cortex, medullary pyramids, and basal ganglia, resulting in a mixed type of CP. Hyperbilirubinemia is associated with a greater risk for the development of dyskinetic CP (OR = 3.589). Unbound bilirubin, when combined with hyperbilirubinemia, can damage the developing central nervous system, especially the basal ganglia and thalamus. This can lead to conditions such as acute and chronic bilirubin encephalopathy and bilirubin-induced neurological dysfunction [19]. Therefore, it is crucial to predict the classification of CP early on the basis of various risk factors to allow timely intervention measures and prevent severe impairment of children's daily living ability.

#### GMFCS

CP is a condition related to upper motor neuron syndrome. It is caused by injury to the nerve centre or the conduction system, leading to excessive release of lower motor neurons, muscle spasm, and overactivity. This is often combined with weakness and low tension in the antagonistic muscles, resulting in an imbalance of muscle activity. The main symptoms are difficulty initiating, adjusting, and maintaining precise movements in the limbs during exercise [26].

The pathological changes in patients with CP are permanent, but motor control disorders can progress, leading to delayed motor development and abnormal postures [27]. The GMFCS is a useful tool for evaluating motor function in individuals with CP. In this study, binary logistic regression analysis was used to assess the correlation between the GMFCS level and risk factors. The study revealed that asphyxia (OR = 1.619) and neonatal convulsions (OR = 1.998) were correlated with more severe motor function impairment (GMFCS level ≥ IV). These findings suggest that these factors can be used to predict the degree of motor function impairment in children with CP. A study from India also indicated that hemiplegic and diplegic CP, as well as a GMFCS classification of ≤ III, predicted less concurrent impairment, but the number of comorbidities increased with greater dysfunction [28]. However, a study from China revealed that [29] there was no relationship between the risk factors for CP in children and the GMFCS level. This may be because the study focused mainly on children registered with the Disabled Persons' Federation. Therefore, further research is needed in the future.

#### Comorbidities

Studies have shown that neonatal seizures are associated with epilepsy (OR = 12.402). HIE (OR = 1.791) and hyperbilirubinemia (OR = 2.328) are related to intelligence level, usually due to abnormal bilirubin metabolism, resulting in brain cell damage and intellectual disability [20]. With respect to hearing, preterm children with CP have a low likelihood of hearing disability (OR = 0.372). The foetus is in the stage of brain nerve centre development in the middle and late stages of pregnancy, and preterm can easily cause central nerve damage. Moreover, premature infants may also have delayed external auditory canal and cochlear nerve development due to congenital malnutrition, resulting in hearing disability [30].

### Effect of the number of risk factors on CP

Approximately 11.2% of children with CP seem to have no known risk factors for CP onset, which is a relatively large portion of the population. This might be due to improved medical care and a decrease in obvious risk factors. However, early intervention can still reduce the occurrence of CP. For these children with CP but no apparent risk factors, genetic mutations may be the cause. This phenomenon is increasingly observed in clinical settings through characteristics such as distinct facial features and unexplained movement disorders, which are often identified through genetic testing. Data indicate that approximately one-third of individuals with CP have an underlying genetic cause, which may overlap with other neurodevelopmental disorders, such as intellectual disability, epilepsy, speech and language disorders, and autism [31]. As shown in Fig. 3, a greater proportion of children with CP who also had epilepsy and intellectual disabilities have no risk factors, which may be due to genetic factors. In the future, we plan to conduct genetic testing on these children to further investigate the factors that impact children with CP.

Studies have shown that CP can be caused by a combination of factors rather than just one single risk factor. It is important to differentiate between associated or risk factors and known causes [32]. For some children with CP, it appears that a series of events, rather than a single occurrence, lead to the development of CP. This concept is known as a "causal path," which refers to a series of interconnected events that ultimately result in the development of a disease. To date, no studies have explored the relationships between the number of risk factors and the classification of CP, the Gross Motor Function Classification System (GMFCS) level, or cooccurring conditions. However, our study indicates that for individuals with three or fewer risk factors, CP is more likely to be associated with both hearing impairment and epilepsy. This could be due to these comorbidities being caused by specific risk factors. On the other hand, in those with more than three risk factors, severe forms of CP (such as spastic quadriplegia and mixed CP), higher GMFCS levels, and intellectual disability were more prevalent. This could be because children with multiple risk factors are more likely to experience extensive brain injury and more severe brain injury.

## Advantages

This study is the first to analyse the risk factors associated with CP in children in Northeast China. It also explored the relationships between the number of risk factors and the classification of CP, the GMFCS level, and comorbidities. The study utilized UpSet diagrams and proportional histograms to illustrate the risk factors present in 536 children with CP. Additionally, logistic regression analysis was employed to investigate potential risk factors that could impact the classification of CP, GMFCS level, and comorbidities.

## Limitations

Initially, we gathered data on the basis of the medical records of numerous individuals in different groups. However, because this was a retrospective study, there might be incomplete medical record information and a lack of documentation for other risk factors. For example, neonatal hypoglycaemia is linked to CP, which impacts executive ability and visual and motor integration [33]. Perinatal ischaemic stroke leads to unilateral brain injury, resulting in spastic hemiplegia [34], which is rarely recorded in our medical and clinical records. Second, as this is a study was conducted in a hospital, it is important to note that the number of children with CP was relatively small. The findings of our study suggest that although hyperbilirubinemia is associated with a 1.635-fold increased risk of hearing disability, this association is not statistically significant [35]. Therefore, further large-scale population-based studies are needed to explore the risk factors for CP in children in Northeast China.

## Conclusion

The majority of children with CP have identifiable risk factors, with the most common being preterm birth or low birth weight. The combination of two risk factors is more prevalent. Analysis of individual risk factors indicated that the risk factors were associated with classification, the GMFCS level, and comorbidities, which aligns with available research. Risk factors are more prevalent in children with severe CP, high GMFCS levels, spastic quadriplegia, and intellectual disability.

## Data Availability

All data generated or analysed during this study will be included in the planned published papers and the supplementary information files.
